# Surface and interface effects in oxygen-deficient SrMnO_3_ thin films grown on SrTiO_3_†

**DOI:** 10.1039/d1cp04998d

**Published:** 2022-01-31

**Authors:** Moloud Kaviani, Ulrich Aschauer

**Affiliations:** Department of Chemistry, Biochemistry and Pharmaceutical Sciences, University of Bern Freiestrasse 3 CH-3012 Bern Switzerland ulrich.aschauer@unibe.ch

## Abstract

Complex oxide functionality, such as ferroelectricity, magnetism or superconductivity is often achieved in epitaxial thin-film geometries. Oxygen vacancies tend to be the dominant type of defect in these materials but a fundamental understanding of their stability and electronic structure has so far mostly been established in the bulk or strained bulk, neglecting interfaces and surfaces present in a thin-film geometry. We investigate here, *via* density functional theory calculations, oxygen vacancies in the model system of a SrMnO_3_ (SMO) thin film grown on a SrTiO_3_ (STO) (001) substrate. Structural and electronic differences compared to bulk SMO result mainly from undercoordination at the film surface. The changed crystal field leads to a depletion of subsurface valence-band states and transfer of this charge to surface Mn atoms, both of which strongly affect the defect chemistry in the film. The result is a strong preference of oxygen vacancies in the surface region compared to deeper layers. Finally, for metastable oxygen vacancies in the substrate, we predict a spatial separation of the defect from its excess charge, the latter being accommodated in the film but close to the substrate boundary. These results show that surface and interface effects lead to significant differences in stability and electronic structure of oxygen vacancies in thin-film geometries compared to the (strained) bulk.

## Abstract 2

The oxygen vacancy formation energy in an SrMnO_3_ thin film grown on a SrTiO_3_ substrate is affected by crystal-field effects at the very surface and the induced electrostatic potential.

## Graphical abstract

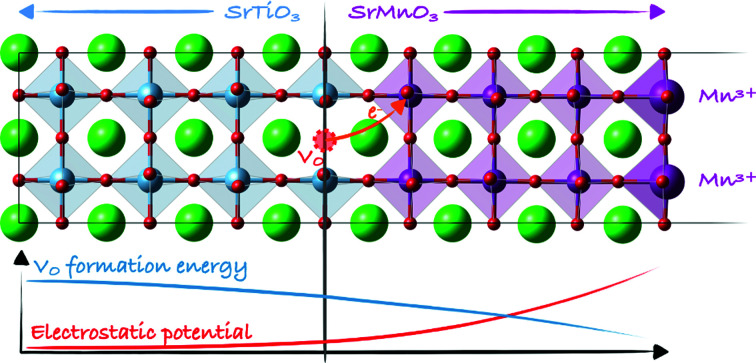

## 1 Introduction

Transition-metal perovskite oxides represent an extremely versatile class of materials that can host a large range of functional properties such as ferroelectricity, magnetism or superconductivity.^1,2^ The emergence of these properties can often be tuned by bi-axial strain, imposed for example by lattice matching during coherent epitaxial growth on a substrate with different lattice parameter.^3^ Compared to bulk perovskite oxides, fundamental changes in properties occur in these thin films. Ferroelectricity and magnetism can, for example, be enhanced^4^ or even introduced in the thin-film material.^5,6^ Moreover, interfaces between the substrate and the film or between different layers of a heterostructure have emerged as an avenue to generate rich and novel electronic phases.^3^

Depending on the synthesis conditions, complex oxides typically contain point defects that can strongly affect conductive, ferroelectric or magnetic properties useful for applications in electronics. While often detrimental to functional properties, defects were also shown to induce novel functionalities in specific cases.^7,8^ Oxygen vacancies (V_O_) are particularly abundant in perovskite oxides under typical synthesis conditions. Depending on their charge state and the localization of the excess charge, V_O_ may lead to changes in oxidation state and local distortions that affect the ferroelectric and magnetic properties.^9–11^ Point-defect engineering could thus be a route to tailor properties for a given application. Our understanding of point defects and their formation energetics and electronic structure is, however, currently mostly limited to idealized bulk or strained bulk systems, neglecting the effect of the substrate–film interface and the film surface, except for select cases like the LAO/STO interface.^12^ Therefore, realistic models containing both surfaces and hetero-interface with the substrate are crucially needed to accurately assess defect-induced phenomena in thin-film systems.

In the present work, we use the model system of a SrMnO_3_ (SMO) thin film grown on a typical (001) TiO_2_-terminated SrTiO_3_ (STO) substrate to study the formation and electronic structure of V_O_ in a thin-film geometry. This model system has recently been experimentally realized, showing excess charge accommodation in the film but close to the interface.^13^ The thermodynamic ground state of SMO is a hexagonal phase,^14^ but it is synthesizable in an orthorhombic perovskite structure when grown on a perovskite structured substrate,^8,15,16^ possibly adopting ordered Brownmillerite phases at high oxygen deficiency.^17^ SMO adopts a G-type antiferromagnetic order with Mn atoms of alternating spin arranged in a 3D checkerboard pattern. Alternatively, this magnetic order can be seen as alternating spins on adjacent Mn-containing (111) planes. V_O_ were previously studied in both materials separately. In SMO and related manganites such as CaMnO_3_ and BaMnO_3_, the three 3d electrons in Mn^4+^ fully occupy the t_2g_ orbitals of majority-spin in the G-type AFM order. Upon V_O_ creation, the e_g_ orbitals of Mn adjacent to the defect are stabilized and accommodate the two excess electrons, resulting in a reduction of both Mn from Mn^4+^ to Mn^3+^.^8,18,19^ This is energetically much preferred over the reduction of a single Mn to Mn^2+^. We note that the oxygen vacancy in this setup is doubly positively charged with respect to the O^2−^ lattice site and charge compensated by the reduction of the two adjacent Mn^4+^ to Mn^3+^. It was also shown that magnetic order and polar distortions can affect the formation energy of V_O_ in these materials.^19,20^ In STO, a large variability in the experimental and theoretical literature reveals that states with excess charge accommodation in the t_2g_ conduction band or in shallow e_g_ defect states are close in energy, results depending also on the crystal structure (cubic or tetragonal) and the size of the simulation cell.^21^ For electrons in the t_2g_ conduction band, the vacancy is doubly positively charged with respect to the O^2−^ lattice site and charge compensated by free electrons, while the vacancy with electrons in e_g_ defect states forms an F-center with the same charge as the O^2−^ lattice site. We note that for SMO, on the other hand, charge localization around an oxygen vacancy does not significantly depend on the cell size and that oxygen-vacancy formation energies computed for one vacancy per 8 formula-unit, 40 atom cell in the related CaMnO_3_^18^ agree well with those observed in experiment.^22^

For the thin-film geometry, our DFT+*U* calculations show that crystal-field changes of the under-coordinated surface atoms lead to a charge transfer towards the surface and hence surface Mn^3+^ species. This asymmetric structure leads to an electric field in this nominally non-polar interface, which strongly affects the defect chemistry. We find that vacancies more easily form at the surface and that the formation energy increases in a near linear fashion with increasing distance from the surface. For oxygen vacancies in the STO substrate, we predict separation of the defect and the charge, the latter residing on Mn atoms at the interface, which leads to a marked reduction in formation energy compared to bulk STO. For our model system, the formation of oxygen vacancies is, therefore, greatly different compared to either of the bulk materials.

## 2 Methods

DFT calculations were performed using Quantum ESPRESSO^23,24^ at the PBE+*U* level of theory^25,26^ with Hubbard *U* calculated self-consistently^27–29^ as 4.26 and 4.48 eV for the Mn and Ti 3d orbitals respectively.^20,21^ All atoms are represented by ultrasoft pseudopotentials^30^ with Sr(4s, 4p, 5s), Mn(3p, 4s, 3d), O(2s, 2p) and Ti(3s, 3p, 3d, 4s) valence electrons. The cutoff for the plane-wave basis was 70 Ry for the kinetic energy combined with 840 Ry for the augmented density.

We model a SMO thin film, grown epitaxially on a (001) STO substrate. Unstrained perovskite SMO has a paraelectric *Pnma* structure with a G-type antiferromagnetic order,^31^ which we use to initialize all calculations throughout this work. The structure is close to the ideal cubic structure with small octahedral tilts and rotations found computationally but not yet observed experimentally.^32^ The STO substrate undergoes, around 105 K,^33,34^ a transition from a high-temperature cubic (space group *Pm*3̄*m*) to a tetragonal antiferrodistortive (AFD, space group *I*4/*mcm*) phase, where TiO_6_ octahedra rotate around the *c*-axis with out-of-phase rotations in consecutive layers (*a*^0^*a*^0^*c*^−^ in Glazer notation^35^). Since our DFT calculations are performed at 0 K, we use the relevant AFD phase of STO.

We construct the STO substrate from the fully relaxed AFD structure as a TiO_2_ terminated 80-atom supercell slab that has 2 × 2 × 4 dimensions compared to the 5-atom cubic cell. The *Pnma* SMO film also has 2 × 2 × 4 dimensions compared to the 5-atom cubic cell and is MnO_2_ terminated. We note that one V_O_ per 160-atom supercell results in a defect concentrations slightly smaller than observed in typical experiments of manganite films.^18,22^ Larger lateral dimensions of the simulation cell lead to negligible changes in formation energies and electronic structure (see Section S4, ESI†). Due to lattice mismatch between STO and SMO, the SMO film experiences tensile strain of about 1.5% when its lattice parameters are adjusted to match the STO substrate. We separate periodic images along the film normal by a 12 Å vacuum and employ a dipole correction in this vacuum layer.^36^ The lowest two atomic layers of the substrate are fixed at bulk positions to mimic the presence of a large and rigid bulk.

The Brillouin zone of this thin-film system is sampled using a 6 × 6 × 1 Monkhorst–Pack^37^*k*-point grid. The convergence criteria for geometry relaxations were 5 × 10^−3^ eV Å^−1^ for forces and 10^−5^ eV for the total energy. Oxygen vacancies can, in principle, occur in multiple charge states when electrons are removed from the cell in addition to the oxygen atom. As shown in Fig. S4 (ESI†), under conditions relevant for materials such as SMO, the charge state without removed electrons dominates. Moreover this charge state is most relevant for oxides that do not contain additional donor or acceptor species that could charge balance ionized oxygen vacancies. The formation energy *E*_f,V_O__ of an oxygen vacancy (V_O_) was calculated as described in ref. 38:

1 *E*_f,V_O__ = *E*_tot,V_O__ − *E*_tot,stoi_ + *μ*_O_,

where *E*_tot,V_O__ and *E*_tot,stoi_ are the total energies of the defective and stoichiometric supercells, respectively and *μ*_O_ is the oxygen chemical potential for which we assume the oxygen-rich limit, evaluated from total energies of water and hydrogen (*μ*_O_ = *E*_H_2_O_ − *E*_H_2__) to avoid the well-known overbinding of O_2_ in DFT.^39^ Since only neutral oxygen atoms were removed from the simulation cell, no terms accounting for the electron chemical potential nor potential alignment are required.

## 3 Results and discussion

### 3.1 Stoichiometric thin film

**Fig. 1:**
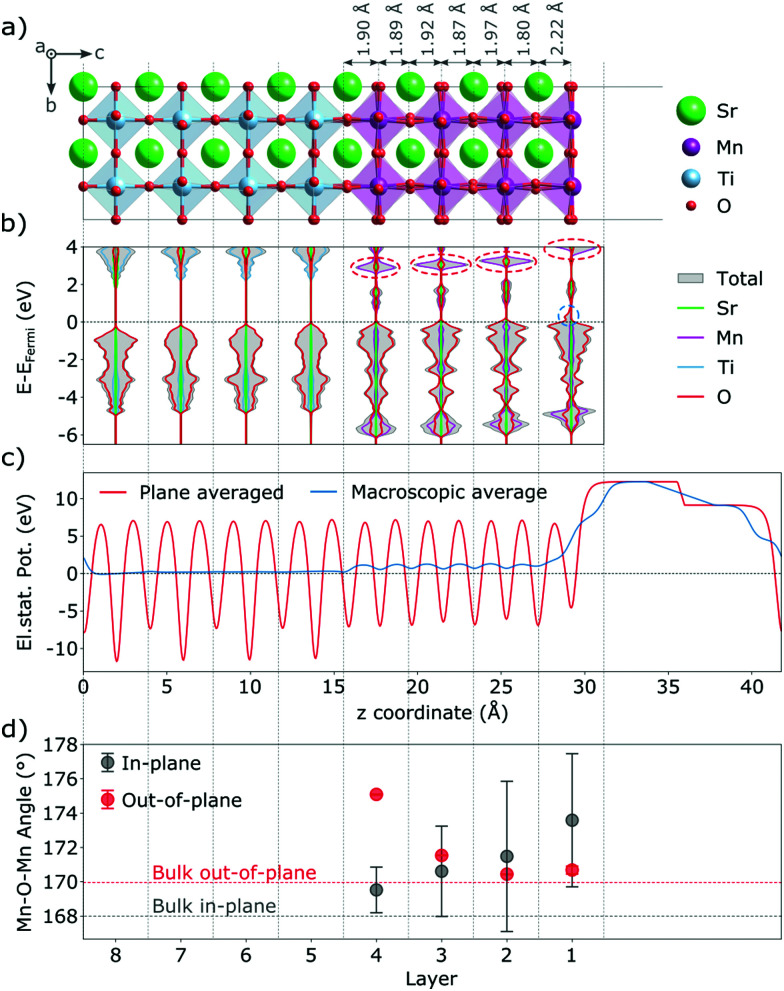
Stoichiometric SMO thin film on a STO substrate: (a) structure and selected interplanar spacings, (b) total and projected layer-resolved density of states (dashed ovals mark features discussed in the text), (c) electrostatic potential and (d) Mn–O–Mn bond angles.

Our thin-film model deviates from SMO bulk in a number of ways that are expected to also affect the behavior and properties of defective films. Due to tensile strain the in-plane (IP) dimensions of SMO are expanded, while the out-of-plane (OP) dimension shrinks according to Poisson's ratio.^40^ This change in lattice parameters is accommodated by changes in Mn–O bond lengths and Mn–O–Mn bond angles (see Fig. 1a and d). In particular the IP Mn–O–Mn angles straighten out at the surface and approach 180°, while the OP bond-angles remain close to the bulk value. We note however that the OP bond-angles are strongly affected at the interface to match the *a*^0^*a*^0^*c*^−^ STO rotation pattern. The *c*/*a* ratio of the film is 0.98, in line with the slight tensile strain.

The truncation of Mn–O bonds at the film surface changes the crystal field of surface Mn atoms from octahedral to approximately square pyramidal. Changes in crystal field were previously shown to affect defect stability in the bulk^41–43^ as well as at surfaces,^44^ while their effect in a thin film geometry was not yet analyzed. The concomitant lowering of Mn 3d energies leads to more favorable electron accommodation at the surface compared to Mn sites in other layers. This change is visible in the layer-resolved projected density of states (Fig. 1b, where at the very surface the characteristic t_2g_ peak visible around 3 eV in lower MnO_2_ layers (circled in red) is significantly destabilized and a small minority-spin (down-polarized Mn) peak is visible just above the Fermi energy (circled in blue). This change in the electronic structure leads to a ferrimagnetic surface layer (see Fig. 1b as well as Fig. S1, ESI† for a narrower energy range around the Fermi level and Fig. S2, ESI† for a comparison of the density of states at deeper layers of the thin film and substrate with bulk SrMnO_3_ and SrTiO_3_).

We evaluated the oxidation state of these surface atoms according to the method proposed in ref. 45, based on the Mn 3d-electron occupation matrix, and found it to be Mn^3+^, while Mn atoms in all other layers retain their nominal Mn^4+^ oxidation state. We verified that the change in oxidation state is not an artefact of the geometry. As shown in Fig. S3 (ESI†), we observe the same change in electronic structure in asymmetric and symmetric SMO surface slabs, indicating that it is an intrinsic feature of MnO_2_-terminated SMO surfaces and not caused by the asymmetry of the setup. Based on the projected layer-resolved densities of states (see Fig. 1b) the electrons leading to surface Mn^3+^ stem from lower lying SMO layers that are slightly electron depleted.

This charge transfer towards the surface has two effects. On one hand, the reduction from Mn^4+^ to Mn^3+^ at the surface causes an increase in ionic radius, which manifests in an expansion of the SrO–MnO_2_ interlayer spacing by about 0.3 Å from 1.91 Å in the bulk to 2.20 Å at the surface, as visible in Fig. 1a). This structural distortion propagates into lower layers, where we observe shorter interlayer spacings below SrO layers (from 1.91 Å to 1.80 Å below the first SrO layer), while those below MnO_2_ layers are expanded. Such a change in geometry will affect the crystal field and could alter the excess charge accommodation upon oxygen vacancy formation. On the other hand, the presence of Mn^3+^ at the surface will repel electrons from the surface. As shown in Fig. 1(c), the change in electrostatic potential is greatest at the surface but a small field exists throughout the whole film. This electric field will lead to excess charges being more favorably accommodated further away from the surface. Nevertheless we note that the SMO film has empty states at lower energies than the STO substrate, which is expected to keep excess-charges in the film rather than the substrate. In the next section, we will investigate how the aforementioned changes in the ionic and electronic structure affect the formation of oxygen vacancies in the thin-film.

### 3.2 Oxygen vacancies in the thin film

**Fig. 2:**
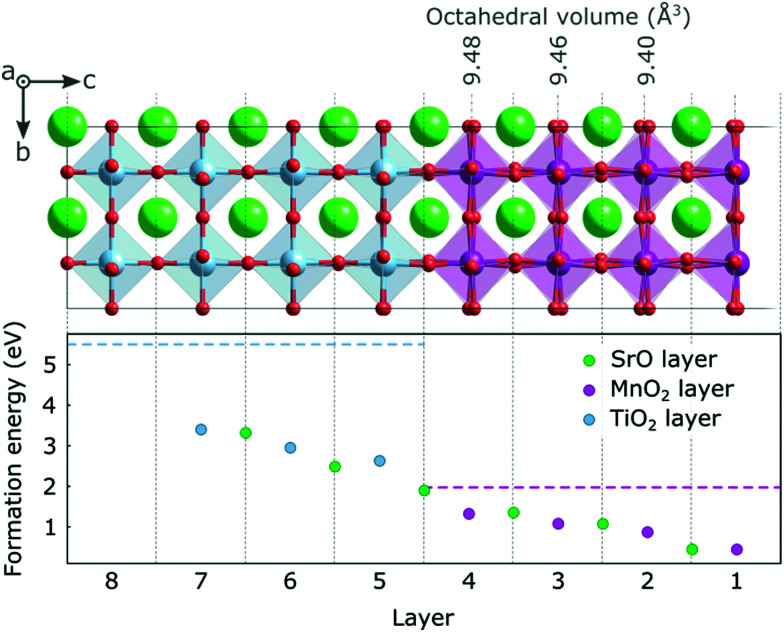
Formation energy for V_O_ under O rich conditions in different layers of the SMO film and the STO substrate. The dashed horizontal lines indicate the theoretical values of the formation energies in 40-atom SMO and STO bulk cells. MnO_6_ octahedral volumes are indicated above the structure.

In Fig. 2 we show the formation energy of oxygen vacancies (V_O_) in the different layers of the thin film. Within each layer, multiple symmetry inequivalent V_O_ positions exist, the variation in formation energy of which is however negligible compared to the effect of the distance from the surface. We observe an increasing trend (by more than 1 eV) in formation energy from the surface to the hetero-interface, which continues into the substrate. This implies that V_O_ will have a tendency to be formed in the surface region, respectively to migrate there, if oxygen mobility is sufficiently high. As indicated by horizontal dashed lines in Fig. 2, it is noteworthy that oxygen vacancy formation in the ultra-thin film is always more favorable than in the bulk, only the SrO layer at the interface reaching the V_O_ formation energy of bulk SMO. The V_O_ formation energy in the substrate, on the other hand, is always lower than in bulk STO. This hints at a different electronic structure of the V_O_ compared to the bulk phases.

**Fig. 3:**
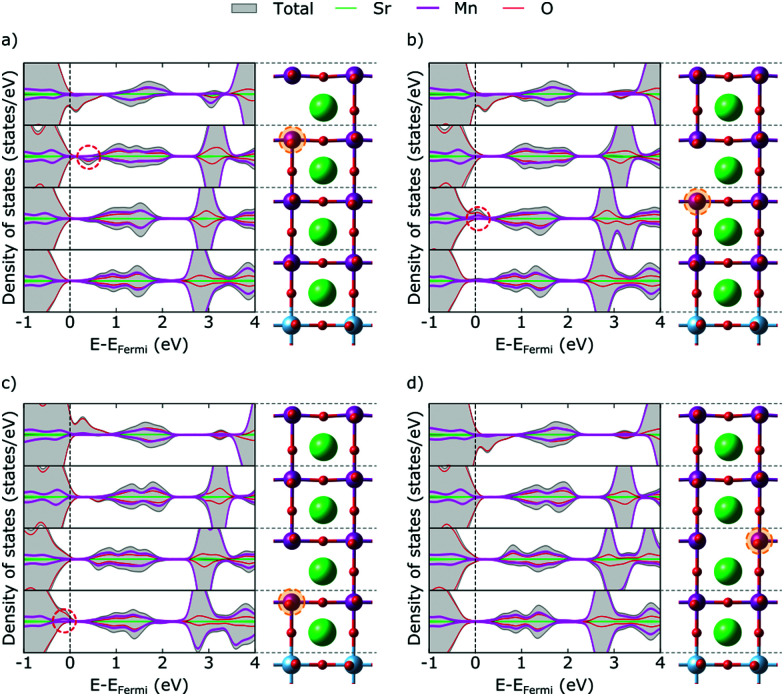
Total and projected layer-resolved density of states for V_O_ (a) in the first, (b) second and (c) third SrO layer and (d) in the third MnO_2_ layer. Dashed ovals mark features discussed in the text. The primarily reduced Mn sites are circled in orange.

The metallic nature of the stoichiometric surface layer greatly complicates the analysis of the electronic structure in presence of a V_O_ and we hence start our discussion with vacancies in the SrO layers, returning to MnO_2_ layers below. In bulk manganites, V_O_ formation results in a lowering of the e_g_ states of the two Mn atoms adjacent to the vacancy, which become the lowest unoccupied states and accommodate the excess electrons resulting from V_O_ formation.^8,18,20^ In the topmost SrO layer of our thin film, we observe a different picture. Instead of localizing on one Mn atom each in the surface and first subsurface MnO_2_ layers, we observe the typical Mn e_g_ defect state only in the first subsurface layer (circled in Fig. 3a). We note that this state remains unoccupied, while we observe filling of the valence-band states, notably in the second layer, that were empty due to the crystal-field changes at the stoichiometric surface. This implies that the charge primarily localizes in the layer below the vacancy, however not in the typical Mn e_g_ defect states.

We see the same pattern also in the second and third SrO layer (Fig. 3b and c), but note that with increasing depth the circled Mn e_g_ defect state becomes occupied while the valence-band states in the first subsurface layer are again empty. We associate this with the slight upwards band bending at the surface induced by the Mn^3+^ ions that causes the defect state in layers further from the surface to be lower in energy than closer to the surface. This is also in agreement with the fact that electrons always localize in layers below the defect, *i.e.* further away from the surface. Moreover, the reduction-less excess-charge accommodation in valence-band states explains the lower formation energy close to the surface, which gradually increases due to increased defect-state occupation in layers further from the surface.

The only exception to this excess-charge accommodation pattern occurs in the SrO layer at the SMO–STO interface, where the V_O_ is formed between a Mn and a Ti atom. Here the reduction occurs on the Mn above the V_O_, which can be rationalized by the lower lying empty Mn states compared to the empty Ti states (see Fig. 1b). This change in electronic structure can account for the marked increase in formation energy for this layer, compared to layers closer to the surface (see Fig. 2).

For V_O_ in the MnO_2_ layers (exemplified for the third MnO_2_ layer in Fig. 3d) we observe an asymmetry of e_g_ peaks in the majority and minority spin channels. Indeed, we observe significantly more Mn e_g_ density of states in the valence band of the vacancy layer than for the other layers. We thus conclude that one of the two Mn sites next to the V_O_ gets reduced (the spin-down Mn in the G-AFM order in this case), whereas the other (the spin-up Mn) does not get reduced, the remaining electron filling the empty valence-band states.

The variation in bond lengths discussed for the stoichiometric surface leads to changes in the octahedral volumes and one could, *via* chemical expansion arguments,^18,46^ expect the V_O_ formation energy to be inversely correlated with the polyhedral volume in the stoichiometric structure. While the strongly expanded truncated polyhedron at the surface is indeed associated with a low formation energy, this trend does not continue towards the interface (see octahedral volumes Fig. 2) since the octahedral volumes increase as does the formation energy. We therefore believe the V_O_ formation energy to be dominated by the band bending induced by the reduced surface Mn^3+^ ions.

### 3.3 Oxygen vacancies in the substrate

**Fig. 4:**
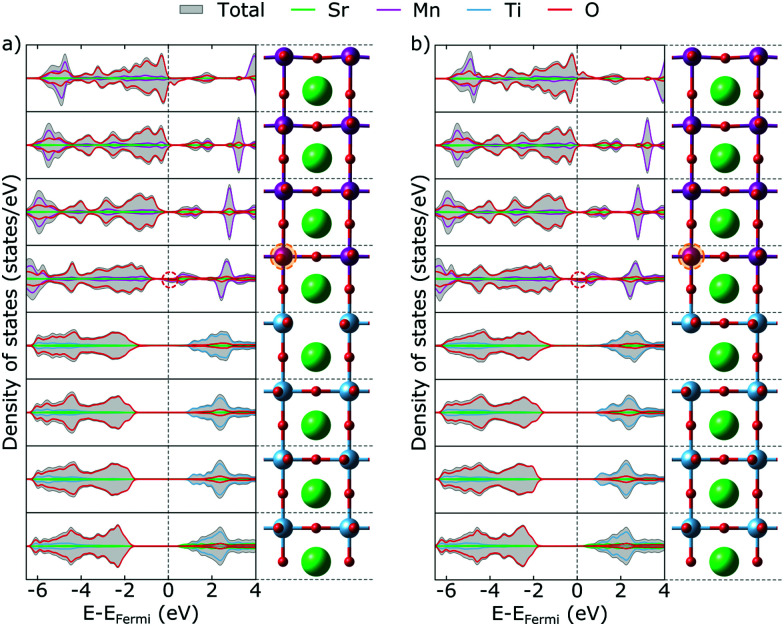
Total and projected layer-resolved density of states for V_O_ (a) in the first TiO_2_ layer and (b) in the first SrO layer of the STO substrate. Dashed ovals mark features discussed in the text. The primarily reduced Mn sites are circled in orange.

In bulk STO, the two excess electrons associated with a V_O_ localize in F-center like states derived from Ti e_g_ orbitals and possibly also populate the conduction band if a spin triplet state is allowed.^9,21,47–49^ In our thin film setup we observe, on the contrary, no localization of electrons in the vicinity of a V_O_ created in the STO substrate. On the contrary both for a V_O_ created in a TiO_2_ or in a SrO layer of the substrate, the excess electrons primarily localize on Mn states at the bottom of the SMO thin film but also fill the above-mentioned valence-band states induced by surface Mn^3+^ (see Fig. 4).

This separation of the V_O_ defect from it's excess charge occurs – at least within the scale of our computational model – independently of the distance of the V_O_ from the interface and can be rationalized by the significant energy difference between empty Mn and Ti states (see Fig. 1). The defect formation energy can be significantly lowered by accommodating electrons in energetically more favorable Mn states than in Ti states that are either in the STO conduction band or just below the conduction band edge.

This interfacial charge transfer is the reason behind the reduced formation energy compared to bulk STO.^21^ Nevertheless we note that V_O_ formation energies will always be significantly larger in the substrate compared to the thin film and that V_O_ will have a driving force for migration from the STO substrate into the SMO thin film.

## 4 Conclusions

In this work, we investigated the effect of the surface and hetero-interface on oxygen vacancies in SrMnO_3_ thin films grown on a SrTiO_3_ substrate. We show that the altered crystal field at a MnO_2_ terminated film surface leads to a charge transfer from valence-band states in lower layers to the surface, forming reduced Mn^3+^ ions at the very surface. This alteration affects oxygen-vacancy formation in the film in two ways. On one hand the holes created in the film due to the above-mentioned charge transfer accommodate some of the excess charge induced by oxygen vacancies. On the other hand, the remaining excess charge that localizes in the vicinity of the vacancy is affected by the surface-Mn^3+^ induced band bending, which leads to formation energies that gradually increase from the surface to the interface. We further show that vacancies in the SrTiO_3_ substrate have larger formation energies compared to the SrMnO_3_ film but transfer electrons to the film, which lowers their formation energy compared to bulk SrTiO_3_.

A previous experimental study^13^ on the same thin film system, used EELS to detect an increased electron density in the MnO_2_ layer closest to the substrate. This was explained based on multiple origins, among them oxygen vacancy formation in the film and a reduction of the substrate under deposition conditions. Based on our findings, oxygen vacancies in the SrMnO_3_ film would have to reside at the very interface to induce charges in that layer. Since there is no driving force for vacancy accumulation close to the interface and since vacancies in other layers would also affect the Mn oxidation state in these layers, oxygen vacancies in SrMnO_3_ seem an unlikely source for the experimental observations. On the other hand, oxygen vacancies in the substrate (likely to be formed under vacuum deposition conditions) would consistently lead to charges in the layer observed by experiment and are a more likely scenario.

Our results show that oxygen vacancy formation in a thin-film geometry can be significantly different from a bulk or strained bulk situation. Not only can the film surface induce subtle changes in the electronic structure that affect excess charge accommodation and stability of oxygen vacancies, but the film can also attract excess electrons from the substrate. These effects are not captured in established strained bulk calculations and are expected to strongly depend on the properties of the film and the substrate. In the present SrMnO_3_ film grown on top of a SrTiO_3_ substrate, these effects lead to a metallic and ferrimagnetic surface with potential applications, in particular, in oxide electronics. We expect similar behavior in other perovskite oxide films with multivalent B-site cations, but note that the different d-orbital occupation can strongly alter the crystal-field effects. Detailed studies on other film materials will be a worthy topic for future studies but we stress that gaining a fundamental understanding of defects in oxide thin films will have to involve surface and interface effects in addition to strain effects.

## Data availability

The main Quantum ESPRESSO pw.x input and output files needed to compute the formation energies in Fig. 2 are available on the Materials Cloud Archive at DOI: 10.24435/materialscloud:1y-q7.

## Conflicts of interest

There are no conflicts to declare.

## Supplementary Material

CP-024-D1CP04998D-s001
