# Supplementary material for: Surface and interface effects in oxygen-deficient SrMnO3 thin films grown on SrTiO3
Source: Phys Chem Chem Phys. 2022 Jan 31;24(6):3951–7. doi: 10.1039/d1cp04998d (PMC8827045; doi:10.1039/d1cp04998d)
Supplement: CP-024-D1CP04998D-s001 [file CP-024-D1CP04998D-s001.pdf]

Electronic Supplementary Material (ESI) for Physical Chemistry Chemical Physics.
This journal is © the Owner Societies 2022

Supplementary information for

Surface and interface effects in oxygen deficient $SrMnO_3$ thin films grown on $SrTiO_3$

Moloud Kaviani and Ulrich Aschauer
Department of Chemistry, Biochemistry and Pharmaceutical Sciences, University of Bern, Freiestrasse 3, CH-3012 Bern, Switzerland

(Dated: January 17, 2022)

S1. ELECTRONIC STRUCTURE OF THE THIN FILM

In Fig. S1a, we show the layer-resolved projected density of states for the SMO portion of the thin-film-substrate system. We focused on a small energy range around the Fermi level as opposed to the broader range shown in Fig. 1a of the main text. This more clearly shows the depletion of the subsurface valence band states and the modification of the surface electronic structure as discussed in the main text.

In Fig. S1b we show, for comparison, the density of states of bulk SMO and the contribution of different d states to the valence and conduction bands respectively.

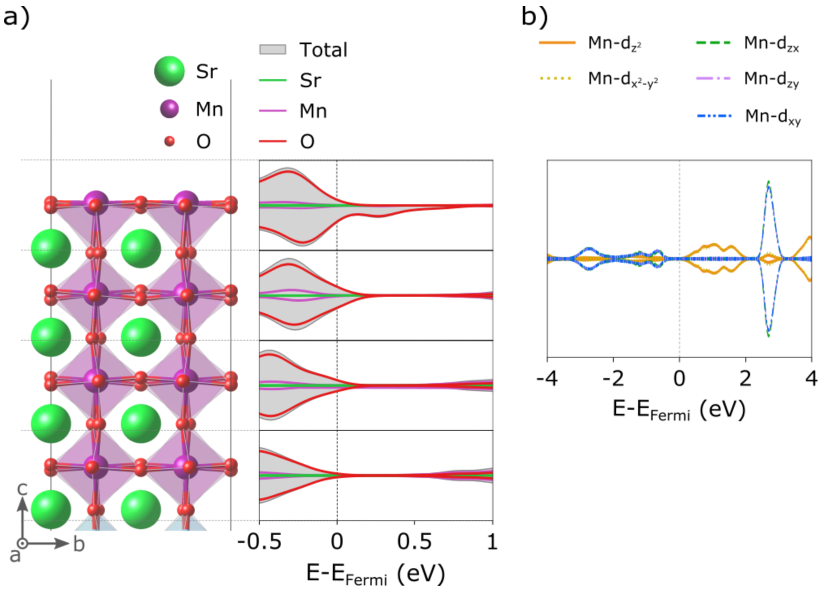

Figure S1. a) Total and projected layer-resolved density of states for the stoichiometric $SrMnO_3$ thin film part of the interface shown in Fig. 1a) of the main text. b) Contribution of different d states to the valence and conduction bands of bulk $SrMnO_3$.

In Figs. S2a) and b) we show the density of states in deeper layers of the SMO film (layer 3 in Fig. 1 in the main text) and the STO substrate (layer 7 in Fig. 1 in the main text) respectively. Our results show that the electronic structure of these layers is virtually unchanged compared to the bulk density of states of the two materials shown in Figs. S2c) and d) respectively.

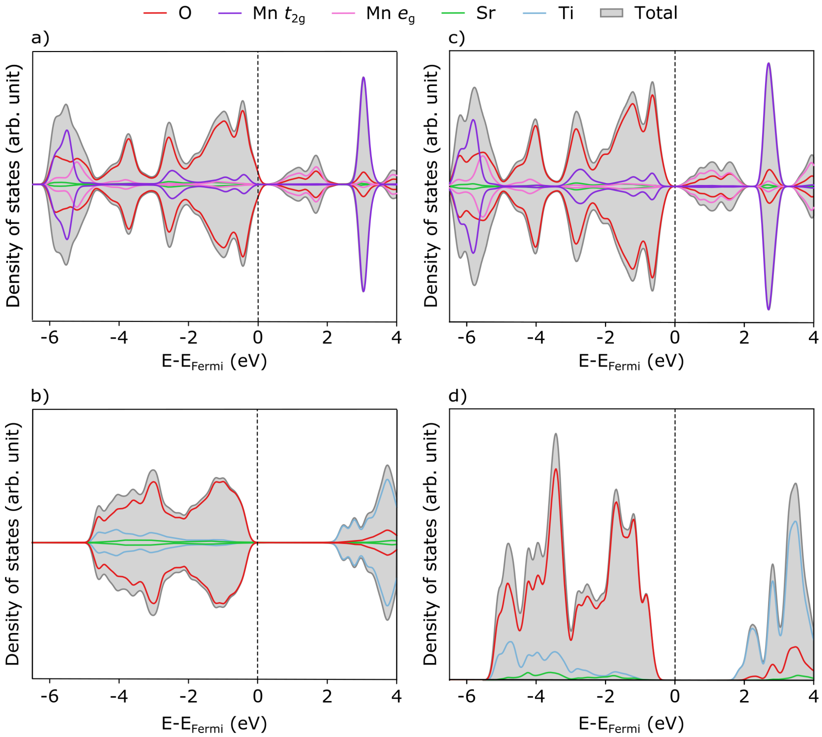

Figure S2. Total and projected density of states for a) the $SrMnO_3$ layer number 3 of the thin film and b) the $SrTiO_3$ layer number 7 of the substrate. Panels c) and d) show the bulk density of states for $SrMnO_3$ and $SrTiO_3$ (non spin-polarized) respectively.

S2. EFFECT OF FILM SYMMETRY

In Fig. S3, we show the layer-resolved projected density of states for an asymmetric $SrMnO_3$ slab with a MnO_2 top surface and a SrO bottom surface as well as for a symmetric slab with two MnO_2 surfaces. The key result is that when both surfaces are MnO_2-terminated and allowed to relax, they develop the same electronic structure as the top surface of the asymmetric slab. Since in the symmetric slab, no spurious electric field due to the presence of two different surface terminations is present, we associate the changed surface electronic structure with crystal-field changes from octahedral to square pyramidal, as mentioned in the main text, rather than it being an artifact associated with the asymmetric geometry of the film.

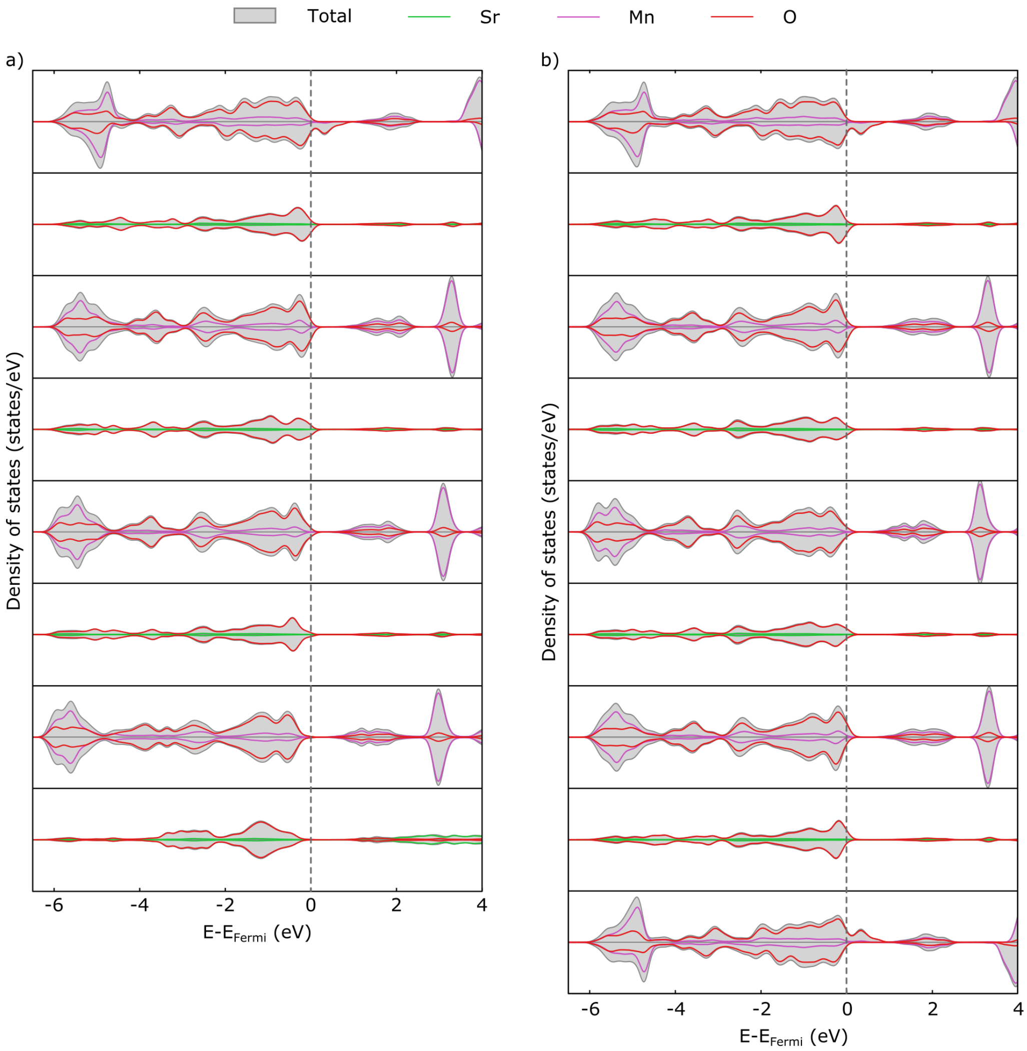

Figure S3. Total and projected layer-resolved density of states for a) the stoichiometric, asymmetric $SrMnO_3$ slab and b) the non-stoichiometric, symmetric $SrMnO_3$ slab.

S3. OXYGEN VACANCY CHARGE STATE

Fig. S4 reports the formation energies of an oxygen vacancy in different layers and for different charge states (neutral = 0, singly = +1 and doubly = +2 ionized). For SMO an n-doped limit with the Fermi energy close to the conduction band edge is most relevant. In this limit, although the 0/+1 charge transition level moves closer to the conduction band edge in deeper layers, the neutral charge state remains most stable for all investigated layers.

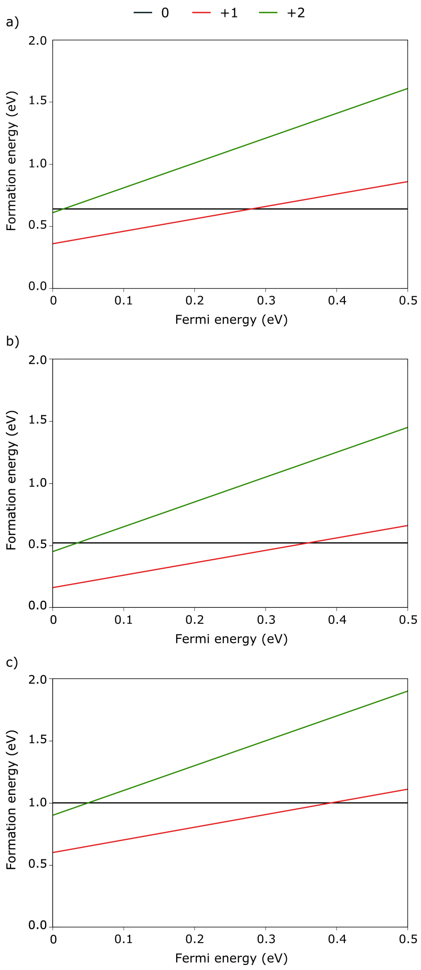

Figure S4. Formation energy of oxygen vacancies in different charge states (neutral = 0, singly = +1 and doubly = +2 ionized) in a) the surface MnO_2 layer, b) the first SrO layer and c) the second MnO_2 layer.

S4. CELL-SIZE EFFECT

To test for cell-size effects, we computed an oxygen vacancy in the top-most SrO layer in a larger 320-atom $\sqrt{2}\times\sqrt{2}\times1$ supercell of the cells used throughout the rest of the manuscript. Reciprocal mesh-sampling was reduced to $4\times4\times1$ in this case and all other computational parameters kept the same. We obtain an oxygen vacancy formation energy of 0.42 eV, which is only 5% smaller than in the smaller cell (0.44 eV), showing that defect-defect interactions are already small for the cells used throughout the manuscript. Moreover, the electronic structure of the defect in the larger cell (see Fig. S5) is very similar to the one in the smaller cell (see Fig. 3a of the main text).

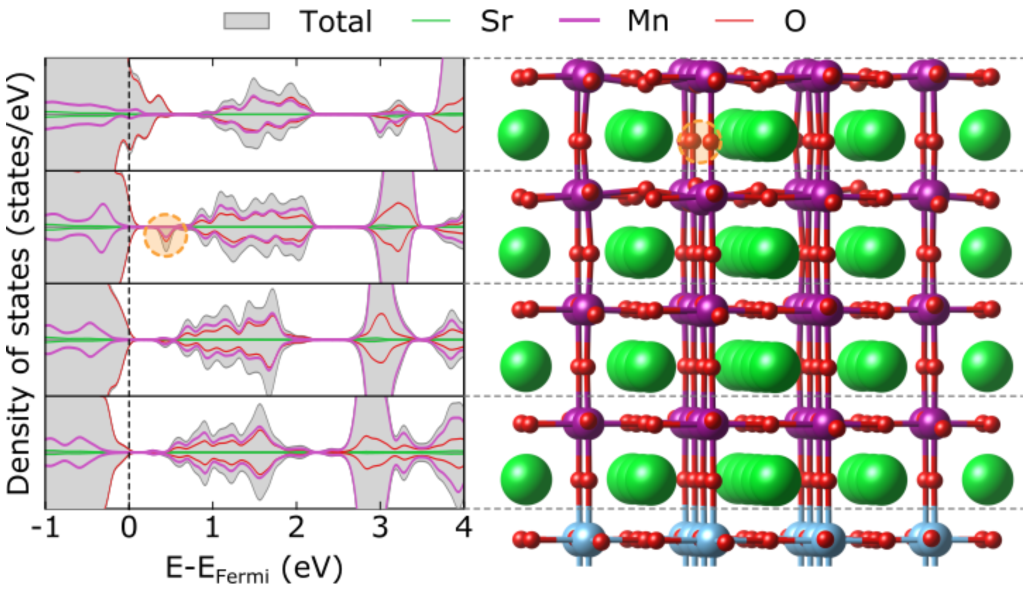

Figure S5. Total and projected layer-resolved density of states for V_O in the first SrO layer computed in a 320-atom $\sqrt{2}\times\sqrt{2}\times1$ supercell of the cells used throughout the rest of the manuscript.
